# Association between Early Childhood Caries and Maternal Factors among 18- to 36-month-old Children in a Rural Area of Cambodia

**DOI:** 10.3290/j.ohpd.a45438

**Published:** 2020-10-27

**Authors:** Yu Kubota, Nhep San Pech, Callum Durward, Hiroshi Ogawa

**Affiliations:** a Assistant Professor, Division of Preventive Dentistry, Faculty of Dentistry and Graduate School of Medical and Dental Sciences, Niigata University, Japan. Study design, conducted survey and data analysis, prepared and edited the manuscript, read and approved the final manuscript.; b Professor, Faculty of Dental Nursing, University of Kampong Cham, Cambodia. Conducted survey and data analysis, read and approved the final manuscript.; c Professor, Faculty of Dentistry, University of Puthisastra, Cambodia. Study design, prepared and edited the manuscript, read and approved the final manuscript.; d Professor, Division of Preventive Dentistry, Faculty of Dentistry and Graduate School of Medical and Dental Sciences, Niigata University, Japan. Study design, read and approved the final manuscript.

**Keywords:** Cambodia, early childhood caries, maternal factors

## Abstract

**Purpose:**

To investigate the associations between early childhood caries (ECC) and maternal factors among 18- to 36-month-old children in one rural province of Cambodia.

**Materials and Methods:**

121 mother-child pairs (male = 67, female = 54; mean age = 25.18 ± 6.24 months) were recruited at several villages in Stueng Trang district, Kampong Cham province. ECC and maternal caries experience were recorded following WHO guidelines. Maternal factors such as literacy and socioeconomic status, as well as child-rearing behaviours, were assessed through an interview questionnaire of the mothers.

**Results:**

ECC and maternal caries prevalence were 54.5% and 84.3%, respectively. Statistically significant associations were found between ECC and: breast-feeding after 18 months; sugary food and beverage intake for the child (p < 0.05); maternal caries experience; illiteracy; night-time breastfeeding, bottle feeding, and late introduction of toothbrushing for the child (p < 0.01). A logistic regression revealed that ECC was more common in children whose mothers had DMFT > 0 (OR = 4.08; 95% CI =1.13-14.75; p = 0.032), children whose mothers were illiterate (OR = 8.21; 95% CI = 1.67-40.85; p = 0.009), children who had night-time breastfeeding after 18 months (OR = 2.76; 95% CI = 1.06-7.19; p = 0.037), and children for whom toothbrushing was introduced after 18 months (OR = 2.87; 95% CI = 1.03-7.97; p = 0.042).

**Conclusion:**

The findings of this study suggest that maternal caries experience and illiteracy, as well as a range of child-rearing behaviours including prolonged night-time breastfeeding and late introduction of toothbrushing were indicators for ECC in this population.

Early childhood caries (ECC) is defined as the presence of one or more decayed (non-cavitated or cavitated lesions), missing (due to caries), or filled tooth surfaces in any primary tooth in a child 71 months of age or younger,^2^ and is associated with a variety of risk factors. Because child oral health is likely to be directly influenced by the mother, maternal factors may play an important role in the development of ECC.^22^ Such mother-related factors may include socioeconomic status (SES), mother’s education, oral health literacy, dietary habits, and frequency of toothbrushing and dental visits. Child-rearing factors which may predispose to ECC include late introduction of toothbrushing habits, certain breast- and bottle-feeding practices, pre-chewing of food for infants (mothers chew food before putting it in their child’s mouth), and high sugar intake in young children.^11^

Primary teeth start to erupt from six months after birth, beginning with the mandibular primary central incisors; the primary dentition is usually complete by the age of three.^1^ Primary tooth eruption and the development of oral function in children are both in a dynamic state during early childhood, and are associated with a change from a totally liquid diet (primarily milk) to a solid-food diet during this period.^3^ Early childhood can also be a period of risk for dental caries, associated with infant feeding practices, poor oral hygiene, and a lack of fluoride.^22^

The first clinical signs of ECC are often seen within the first two years of life, initially affecting the maxillary primary central incisors.^10^ In Cambodia, ECC is a significant health issue affecting almost every child; its prevalence and severity are higher than in all other countries in Southeast Asia.^8^ A recent study conducted in a rural area of Cambodia showed that the prevalence of ECC was 30% among 1-year-olds and 71% among 2-year-olds.^12^ Another recent survey of ECC in three Cambodian provinces found a prevalence of 10% among 1-year-old children, and 84% among those aged three years.^24^ The National Oral Health Survey in 2011 found a mean dmft of 9.0 and a prevalence of 93% among 6-year-olds.^6^ Authors of these studies have recommended that ECC prevention should focus both on mothers and their preschool children and should start early, preferably during pregnancy.

A previous study on maternal factors and ECC in Cambodia showed that high caries experience in mothers was statistically significantly associated with the presence of ECC in their children.^25^ However, this study targeted children whose mean age was 42 months, and who had mostly attained their complete primary dentition. The study focused on caries and did not explore a wide range of related maternal factors. The purpose of the present cross-sectional study was to investigate the associations between ECC and maternal factors among 18- to 36-month-old children in a rural area of Cambodia.

## Materials and Methods

### Ethical Approval and Informed Consent

This cross-sectional study was conducted according to the guidelines of the Declaration in Helsinki and ethical approval was obtained from the Ethics Committee of Niigata University (2017-0187). Before the study commenced, permission was sought from the local health administrative district in Cambodia and community leaders were consulted. Each participant was provided with an information sheet. The purposes and processes of the study were explained and written informed consent was obtained from each mother.

### Study Site and Participants

The study was conducted in Steung Trang district, Kampong Cham province from May 2018 to January 2019, Cambodia. 121 mother-child (aged 18- to 36-months) pairs (males = 67, females = 54; mean age = 25.18 ± 6.24 months), who visited the Khpob Ta Nguon Health Center in Khpob Ta Nguon commune (small administrative district) to receive health check-ups, vaccinations and medicine, were enrolled in the study.

The ages of the child participants were confirmed by referring to the birth registration records in the Health Center and their maternal health booklets. Mother-child pairs were randomly recruited by the chiefs in each village. Mothers who were edentulous, who declined consent, or who were not the main caregivers for the children were excluded, as were children with fewer than eight erupted teeth and children with a major disability.

### Oral Examination

Before the oral examinations commenced, intra-examiner calibration was carried out on 20 subjects by one trained dentist. A kappa score of 0.87 was obtained, indicating a high level of agreement. The oral examinations were conducted at the Khpob Ta Nguon Health Center by visual inspection under natural light following the WHO Oral Health Surveys Basic Methods.^32^ Mothers’ caries and the ECC status of the child were assessed in a sitting or a knee-to-knee supine position, respectively. DMFT/dmft scores were calculated correspondingly.

### Questionnaire Interview

Before interviewing the mothers in the study, preliminary interviews of 20 additional mothers by three local, trained interviewers were carried out to verify the validity and reliability of the questionnaire. The Cronbach’s coefficient score of 0.77 indicated that internal consistency was acceptable. The questionnaire included questions on sociodemographic background, mother-related factors and child-rearing practices, including dietary and oral hygiene practices related to ECC.

### Statistical Analysis

Data were entered into an Excel spreadsheet and transferred to the statistical software package SPSS (version 25.0, IBM; Armonk, NY, USA) for analysis. Subgroup comparisons of mean DMFT and dmft scores were performed using the Mann-Whitney and Kruskal-Wallis tests. The chi-squared test was used to investigate associations between variables and ECC. A logistic regression analysis of ECC was performed to determine the predictors of ECC with a step-wide method by selecting the variables with statistically significant p-values and with less than 0.1 in the univariate analysis. Statistical significance was set at p < 0.05.

## Results

The caries prevalence observed for mothers was 84.3%, and the mean DMFT, DT, MT, FT scores were 6.59 ± 6.04, 3.13 ± 2.80, 1.50 ± 2.22, and 1.98 ± 3.57, respectively. Among the children, ECC prevalence was 54.5%, and the mean dmft score was 2.81 ± 3.49. All teeth with ECC were untreated, and no statistically significant differences were observed between males and females. ECC prevalence and the mean dmft scores increased with age (p < 0.01) (Table 1).

**Table 1:** Caries/ECC prevalence, mean DMFT/dmft among mother-child pair (n = 121)

Variable	Number (%)	Caries prevalence (%)	p-value	DMFT (mean ± SD)	p-value	DT (mean ± SD)	MT (mean ± SD)	FT (mean ± SD)
**Mothers**	121 (100)	102 (84.3)	–	6.59 ± 6.04	–	3.13 ± 2.80	1.50 ± 2.22	1.98 ± 3.57
Variable	Number (%)	ECC prevalence (%)	p-value	dmft (mean ± SD)	p-value	d (mean ± SD)	m (mean ± SD)	f (mean ± SD)
**Children**								
Males	67 (55.4)	40 (59.7)	0.21	3.13 ± 3.80	0.32	3.13 ± 3.80	0	0
Females	54 (44.6)	26 (48.1)		2.41 ± 3.06		2.41 ± 3.06	0	0
18–23 months	55 (45.4)	21 (38.2)	0.001	1.60 ± 2.48	< 0.001	1.60 ± 2.48	0	0
24–39 months	31 (25.6)	17 (54.8)		3.16 ± 4.16		3.16 ± 4.16	0	0
30–36 months	35 (29.0)	28 (80.0)		4.40 ± 3.60		4.40 ± 3.60	0	0
Total	121 (100)	66 (54.5)		2.81 ± 3.49		2.81 ± 3.49	0	0

Mothers: caries, DMFT; children: ECC, dmft.

Among mother-related factors, literacy and DMFT showed statistically significant associations with both ECC and dmft prevalence of the children (p < 0.01). Children of mothers with DMFT > 0 had a statistically significantly higher ECC prevalence and dmft score (3.2 and 4.9 times, respectively) than those whose mothers had no caries experience (Table 2).

**Table 2:** Association between ECC and mother-related factors (n = 121)

Variables	Number (%)	ECC prevalence (%)	p-value	dmft (mean ± SD)	p-value
**Literacy (Can read and write)**					
Yes	102 (84.3)	49 (48.0)	0.001	2.46 ± 3.46	0.001
No	19 (15.7)	17 (84.5)		4.68 ± 3.16	
**Homemaker**					
Yes	34 (28.1)	14 (41.2)	0.065	2.21 ± 3.17	0.15
No	87 (71.9)	52 (59.8)		3.05 ± 3.60	
**DMFT**					
0	19 (15.7)	4 (21.1)	0.002	0.74 ± 1.52	0.003
1–6	55 (45.5)	30 (54.5)		2.78 ± 3.36	
7–22	47 (38.8)	32 (68.1)		3.68 ± 3.89	
**Sugary food intake**					
Once a day or more	46 (38.0)	25 (54.3)	0.82	2.89 ± 3.59	0.94
Several times a week	57 (47.1)	30 (52.6)		2.70 ± 3.40	
Never	18 (14.9)	11 (61.1)		2.94 ± 3.73	
**Sugary beverage intake**					
Once a day or more	51 (42.1)	30 (58.8)	0.65	3.14 ± 3.57	0.52
Several times a week	45 (37.2)	24 (53.3)		2.27 ± 2.97	
Never	25 (20.7)	12 (48.0)		3.12 ± 4.17	
**Toothbrushing**					
Three times	39 (33.2)	16 (41.0)	0.062	2.46 ± 3.79	0.18
Twice	76 (62.8)	45 (59.2)		2.89 ± 3.40	
Once or never	6 (5.0)	5 (83.3)		4.00 ± 2.82	
**Alcohol intake**					
Yes	32 (26.4)	18 (56.3)	0.82	3.00 ± 3.54	0.64
No	89 (73.6)	48 (53.9)		2.74 ± 3.49	


Among child-rearing factors, ECC prevalence was statistically significantly associated with breastfeeding after 18 months, frequent sugary food and beverage intake for the child (p < 0.05), night-time breastfeeding after 18 months, bottle-feeding after 18 months and the introduction of toothbrushing after 18 months (p < 0.01). Bottle-feeding, night-time breastfeeding after 18 months, the introduction of toothbrushing after 18 months (p < 0.05), and frequent sugary food and beverage intake by children (p < 0.01) were statistically significantly associated with dmft. Children who started toothbrushing after 18 months had 2.2 and 3.3 times higher ECC prevalence and dmft than those who started brushing at a younger age, respectively. 83.5% of mothers agreed that breastfeeding at night helps a child to relax and sleep (Table 3).

**Table 3:** Association between ECC and child-rearing factors (n = 121)

Variables	Number (%)	ECC prevalence (%)	p-value	dmft (mean ± SD)	p-value
**Breastfeeding**					
≤18 months	51 (42.1)	22 (43.1)	0.031	2.63 ± 3.96	0.14
> 18 months	70 (57.9)	44 (62.9)		2.94 ± 3.14	
**Night-time breastfeeding**					
≤18 months	48 (39.7)	17 (35.4)	0.001	1.87 ± 3.51	0.001
>18 months	73 (60.3)	49 (67.1)		3.42 ± 3.37	
**Bottle feeding**					
≤18 months	51 (42.1)	31 (44.3)	0.008	2.34 ± 3.45	0.022
>18 months	70 (57.9)	35 (68.6)		3.45± 3.48	
**Do you agree that breastfeeding at night can help a child relax and sleep?**					
Yes	101 (83.5)	54 (53.5)	0.59	2.45 ± 3.00	0.13
No	20 (16.5)	12 (60.0)		4.65 ± 5.06	
**Introduction of toothbrushing**					
≤18 months	35 (28.9)	10 (28.6)	< 0.001	1.06 ± 2.33	< 0.001
>18 months	86 (71.1)	56 (65.1)		3.52 ± 3.64	
**Sugary food intake for child**					
Once a day or more	45 (37.2)	32 (71.1)	0.019	3.84 ± 3.58	0.009
Several times a week	49 (40.5)	22 (44.9)		2.06 ± 3.09	
Never	27 (22.3)	12 (44.4)		2.44 ± 3.74	
**Sugary beverage intake for child**					
Once a day or more	44 (36.4)	31 (70.5)	0.023	4.23 ± 4.05	0.004
Several times a week	43 (35.5)	21 (48.8)		2.19 ± 2.90	
Never	34 (28.1)	14 (41.2)		1.76 ± 2.82	
**Pre-chewing**					
Yes	23 (19.0)	16 (69.6)	0.11	4.30 ± 4.37	0.062
No	98 (81.0)	50 (51.0)		2.46 ± 3.18	


In a logistic regression analysis adjusted for children’s sex, age, and number of erupted primary teeth, children of mothers with DMFT > 0 and children whose mothers were illiterate were 4.08 and 8.21 times more likely to develop ECC, respectively. Among child-rearing factors, children who continued night-time breastfeeding after 18 months and those who started toothbrushing after 18 months of age were 2.76 and 2.87 times more likely to have ECC than other children (Table 4).

**Table 4:** Logistic regression analysis on early childhood caries (n = 121)

Independent variables	Dependent variable: early childhood caries (0: no, 1: yes)			
	S.E.	p-value	Odds	95% CI
**Mother’s caries**				
0: no (ref)	0.65	0.032	4.08	1.13 – 14.75
1: yes				
**Literacy**				
0: yes (Ref)	0.81	0.009	8.21	1.67 – 40.85
1: no				
**Night-time breastfeeding after 18 months**				
0: no (ref)	0.47	0.037	2.76	1.06 – 7.19
1: yes				
**Toothbrushing by 18 months**				
0: yes (ref)	0.52	0.042	2.87	1.03 – 7.97
1: no				

Adjusted for sex and children’s age and tooth number.

## Discussion

In the present study, we investigated the associations between ECC and maternal factors among 18- to 36-month-old children in a rural area of Cambodia. The prevalence and severity of caries among these Cambodian mothers was high and mostly untreated. The prevalence and severity of ECC among the children was also high and increased rapidly with age. These findings were consistent with findings from several previous Cambodian studies.^12^,^24^ No children had received dental treatment.

In previous Cambodian and other studies of mothers and preschool children, children of mothers without caries experience were statistically significantly less likely to have ECC.^25^,^28^,^29^ The present study found a similarly strong association between ECC and maternal caries experience. This association between the caries status of mothers and their children should be investigated further in longitudinal studies; it serves as an important predictor of which children are at a higher risk of developing ECC.

In this study, 15.7% of mothers were illiterate, and there was a clear positive association between maternal illiteracy and ECC. A similar finding was observed in the National Oral Health Survey in Iran.^9^ In rural Cambodia, it is known that many girls drop out of school at a young age in order to earn money for the family (e.g. in a factory or farm) or to take care of their siblings. Government reports have demonstrated an increase in enrollment in primary schools in Cambodia in recent years,^13^ and UNESCO in 2015 reported a literacy rate among the population aged 15 years and older increased from 67% to 80% over the past 20 years.^27^ However, illiteracy is still at an unacceptable level among Cambodian women and girls, and remains a challenging issue for the Cambodia government. Illiteracy is known to be associated with a range of poor health outcomes and social disadvantages, and the present study lends supports to this.^16^,^17^ Cambodian women who are illiterate will have less opportunity to learn from written materials, including health education materials. They are more likely to have left school at a young age, and therefore may not have had the opportunity to learn about good nutrition, hygiene, and recommended child-rearing practices from school health lessons. Thus, improving the literacy of mothers might have an impact on the oral health of their children.

Although statistically significant differences were not observed, the children whose mothers were homemakers had a lower prevalence of ECC than children whose mothers worked outside of the home. In recent decades in Cambodia, many families have moved from rural to urban areas, and a large proportion of mothers have obtained work in factories, leaving their children to be looked after during the day by other caregivers, such as grandparents or other relatives.^7^ It is believed that many of these caregivers may be provide children with feeding bottles and sweet snacks during the day. 57.9% of the children in this study were still using the bottle after 18 months of age. These dietary habits may be contributing to the high level of ECC seen in this population. Since many young children are cared for during the day by grandparents and other family members, oral health education to prevent ECC should also be targeted at these caregivers and not only the mothers.

The transition from exclusive breastfeeding to solid foods – referred to as complementary feeding or weaning – typically covers the period from 6-23 months of age, even though breastfeeding may continue beyond this. Breastfeeding for up to two years is recommended as it helps prevent infection and provides important nutrients for growing child.^30^ On the other hand, prolonged breastfeeding may contribute to nutritional imbalance and in some cases ECC.^15^

A systematic review that aimed to summarise the evidence about the relationship between breastfeeding and dental caries concluded that frequent breastfeeding beyond 12 months of age, in particular nocturnal breastfeeding, was associated with an increased risk of ECC.^21^

In this study, the majority of children continued breastfeeding, including during the night, for more than 18 months, and this was associated with a statistically significantly higher prevalence of ECC. More than 80% of mothers agreed that breastfeeding at night helped a child to relax and sleep, indicating the positive views Cambodian mothers have about this widespread practice. Almost all mothers in Cambodia sleep with their infants, often for several years. On the other hand, they were likely to have lower dmft scores than those without it, although statistically significant differences were not found. Breast milk has been shown in vitro to be more cariogenic than cow’s milk because of the higher content of lactose.^4^ Breast milk consumed during the night often remains in the mouth for a long time and can be broken down by cariogenic plaque bacteria to produce demineralisation and later cavitation on the surfaces of the erupted primary teeth, initially on maxillary incisors. Bottle-feeding, which was also highly prevalent in this study, can have the same effect. Oral health outcomes have been shown to be associated with knowledge, attitudes and behaviours, and these factors may be associated each other.^18^ In the present study, we did not investigate mothers’ knowledge about the influence prolonged night-time breastfeeding or bottle-feeding might have on ECC. This could be an area of our future research.

Recently in Cambodia, because of steady economic growth and urbanisation, lifestyles have been changing, with an increase in sugar consumption observed among all age groups.^19^ In the present study, children who had a higher sugary food and beverage intake were likely to have higher ECC and a higher dmft. On the other hand, approximately 25% of children under age five have been documented as undernourished.^5^ Therefore, the recommendations on weaning practices, including nutritional management and sugar control, should be followed from an early age. All of these factors need to be addressed in order to improve this situation, and the solution lies outside of the present oral health-care system in Cambodia. An integrated approach involving maternal child health services and local village health providers may have more chance of success.

The International Association for Paediatric Dentistry (IAPD) recommends that caregivers start brushing the <3-year-old child’s teeth with a smear-sized amount of fluoride toothpaste twice a day as soon as the primary incisors erupt.^22^ However, our study found that only 28.9% of mothers started toothbrushing before 18 months when the primary first molars erupt, and that the late introduction of brushing was a risk predictor for ECC. These findings were similar to a recent study among older preschool children in several provinces of Cambodia.^26^ Although the present study did not ask whether a fluoride toothpaste had been used when brushing, fluoride in toothpaste has been shown to be important in providing protection against caries. Fortunately, its use appears to be increasing across Cambodia, although many toothpastes marketed for children contain suboptimal levels of fluoride. Use of a small amount of 1000 ppm fluoride toothpaste twice daily should be recommended for preschool children in Cambodia, which has no fluoridated water supplies.^20^,^32^

In rural Cambodia, lack of oral health personnel is one of the other important issues when considering which interventions might be possible and appropriate to address the ECC problem. Residents in many rural areas have difficultly accessing dental services, and there are very few schools with any type of oral health program. In addition, most dentists are located in the main cities with few residing in rural areas. It is clear from the present study, as well as other recent Cambodian studies, that interventions which begin when children reach school are too late to prevent most primary tooth decay. Therefore, mothers and preschool children should be targeted during pregnancy and preschool years. Interventions to promote oral health should involve not only dental personnel, but other health workers, such as midwives, nurses and health volunteers who routinely see pregnant women and infants, and have an opportunity to intervene, for example, during vaccination visits. In Steung Trang district, health volunteers are assigned to every community and are responsible for monitoring and promoting health and addressing health inequalities in rural areas.^14^ To date, however, only one oral health program involving nurses, midwives and health volunteers has been initiated in Cambodia. Initial results have been positive.^23^ Engaging such personnel to promote the oral health of mothers and young children presents an important opportunity.

The strengths of this study included having only examiner, the high reliability scores of the examiner and interviewers, and the ability to record the exact age of each child, thanks to the availability of accurate birth registration data. There was also a good range of sociodemographic backgrounds of participants. On the other hand, the relatively small number of participants and the cross-sectional nature of the study were limitations, and therefore causal relationships between ECC and associated factors could not be established. Since we did not record the presence of non-cavitated lesions during the oral examinations, this may have resulted in an underestimation of the prevalence and severity of ECC. In addition, only mothers who participated in maternal health check-ups in the district were included in this study. These mothers may have had a higher level of concern for their child’s health compared to other mothers in the community, resulting in selection bias.

## Conclusion

This group of rural Cambodian mothers and their preschool children had high caries experience with little access to dental treatment. Maternal caries experience, maternal illiteracy and the late introduction of toothbrushing were indicators for ECC in their children. In rural Cambodia, Health Centers play a significant role in providing a variety of basic medical services, such as antenatal visits, vaccinations and regular health check-ups for mothers and their children, whereas dental services are still largely neglected. Oral health programs integrated with other health services targeting mothers and preschool children should be conducted in order to address the ECC problem in Cambodia.
